# Supplementary material for: Defucosylation of Tumor-Specific Humanized Anti-MUC1 Monoclonal Antibody Enhances NK Cell-Mediated Anti-Tumor Cell Cytotoxicity
Source: Cancers (Basel). 2021 May 25;13(11):2579. doi: 10.3390/cancers13112579 (PMC8197514; doi:10.3390/cancers13112579)
Supplement: Supplementary file 1 [file cancers-13-02579-s001.zip › cancers-1190921-supplementary.pdf]

cancers

MDPI

Article

Defucosylation of Tumor-Specific Humanized Anti-MUC1 Monoclonal Antibody Enhances NK Cell-Mediated Anti-Tumor Cell Cytotoxicity

Ying Gong, Roel G. J. Klein Wolterink, Valeriia Gulaia, Silvie Cloosen, Femke A. I. Ehlers, Lotte Wieten, Yvo F. Graus, Gerard M. J. Bos, and Wilfred T. V. Germeraad

Supplementary Materials

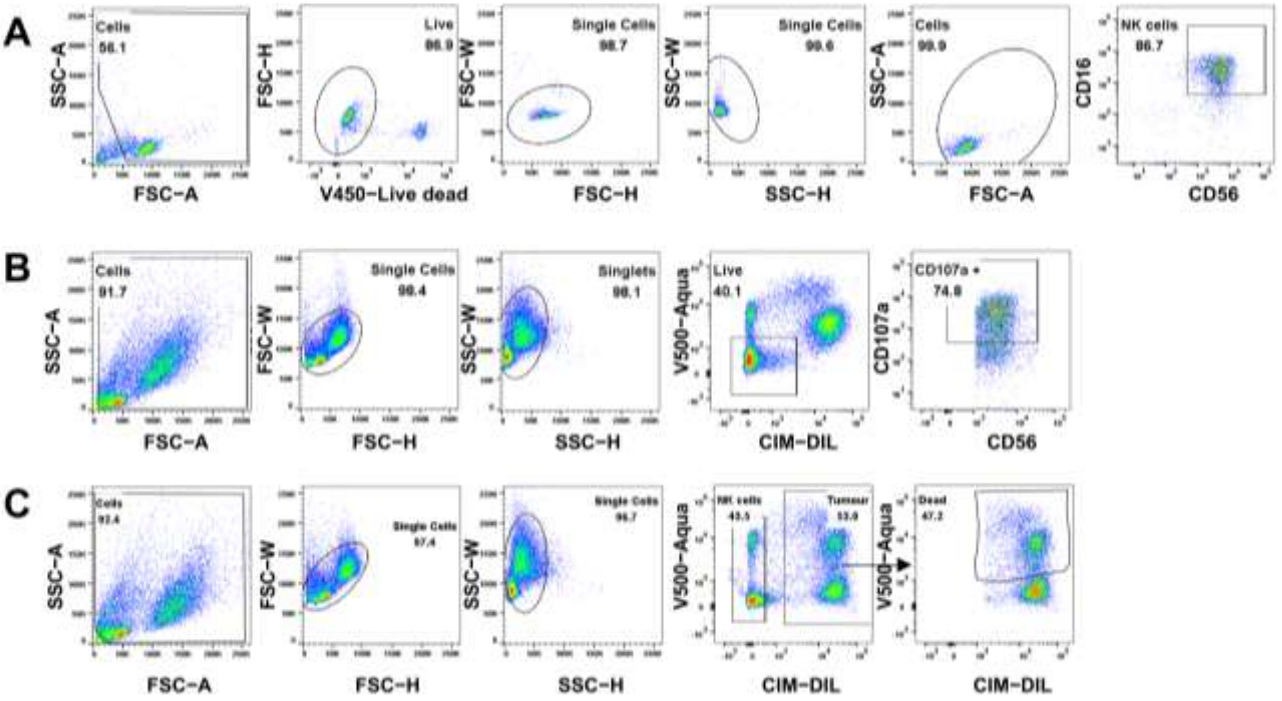

Figure S1. Flow cytometric analysis of NK cell purity, degranulation and cytotoxicity. (**A**) Representative sample of flow cytometric analysis of NK cell purity and CD16 expression after NK cell isolation using negative selection. (**B**) Example of flow cytometric analysis of NK cell degranulation, measured as CD107a expression. (**C**) Example of flow cytometric analysis of NK cell cytotoxicity, measured as the percentage of tumor cell death. Tumor cells were labeled with the dye CIM-DIL. Numbers in plots indicate gate frequencies.

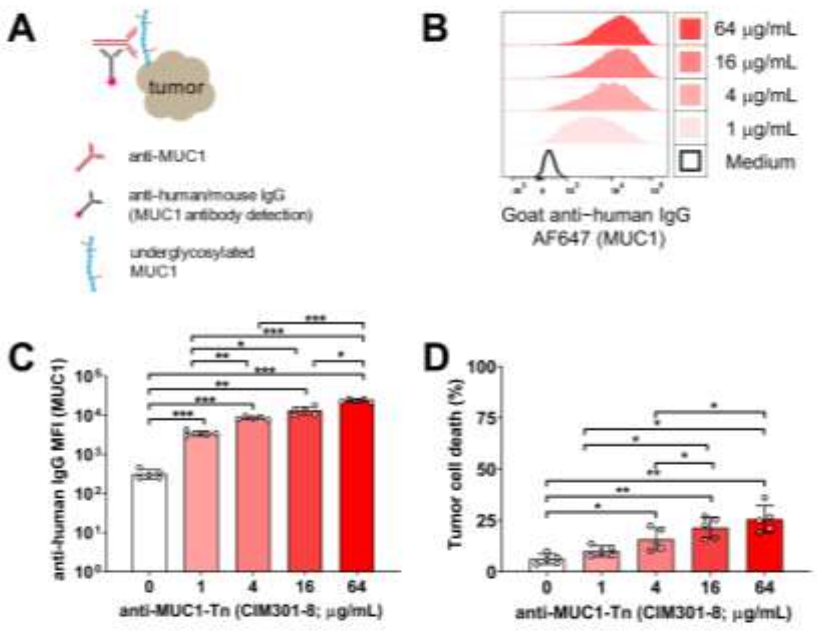

Figure S2. Higher concentrations of humanized anti-MUC1 antibodies induce Jurkat cell death. (**A**) Schematic overview of the analysis of the effects of increasing concentration of defucosylated CIM301-8 anti-MUC1-Tn/STn antibodies on Jurkat tumor cells. (**B**) Overlay histogram showing binding of anti-MUC1 antibodies (CIM301-8; defucosylated Fc-tail) to Jurkat cells, detected using a labeled anti-human IgG antibody. One representative sample is shown. (**C**) Quantification of anti-MUC1 CIM301-8 binding to Jurkat cells as described in B, expressed as median fluorescence index. (**D**) Quantification of anti-MUC1-induced cell death of Jurkat cells after incubation with increasing concentrations of CIM301-8 antibodies (without NK cells). Differences between groups were calculated using one-way ANOVA with Tukey's multiple comparisons test. Not significant (n.s.); $p < 0.05$ (*); $p < 0.01$ (**); $p < 0.0001$ (***).

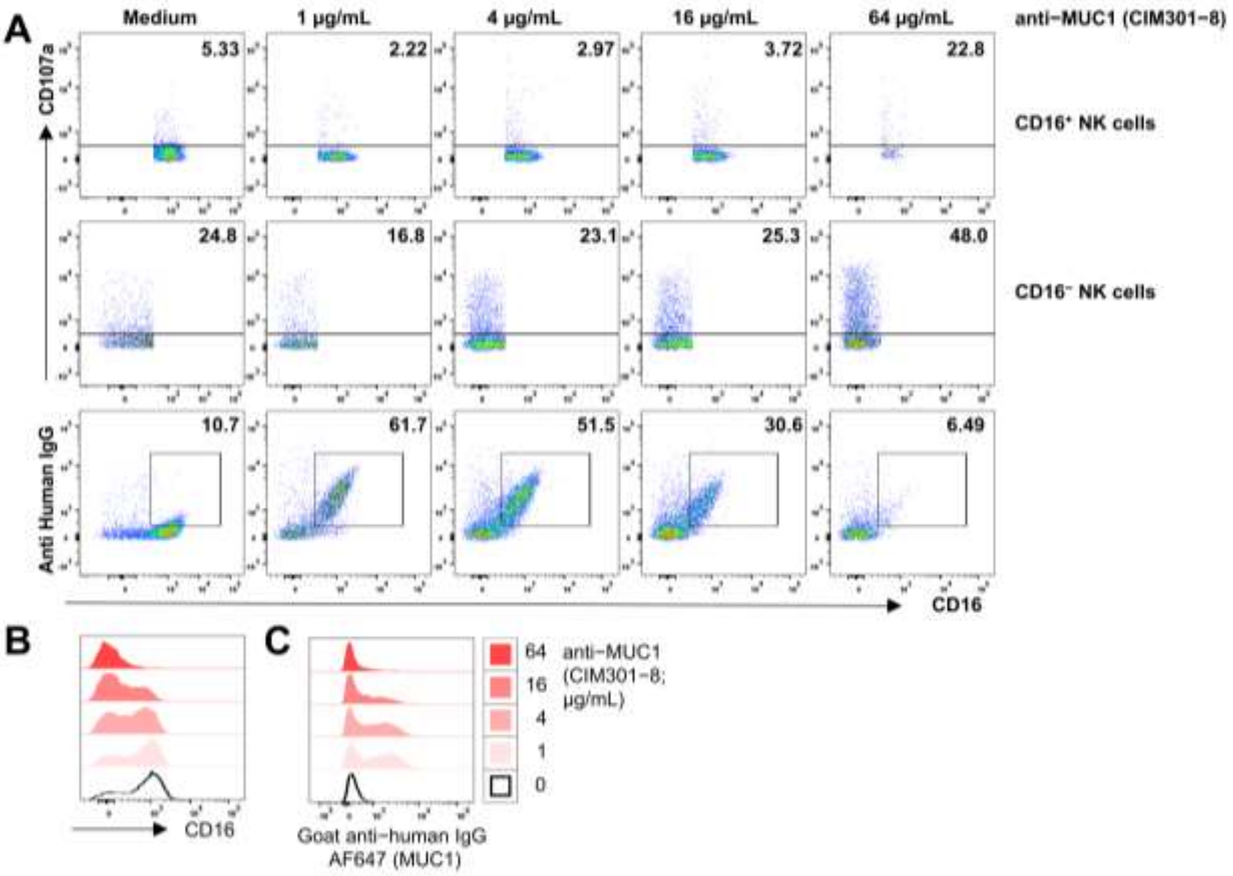

Figure S3. Increasing concentrations of humanized anti-MUC1 antibodies activate NK cells and induce down-regulation of CD16 expression. (**A**) Representative flow cytometry dot plots of CD107a expression on CD16 $^{+}$ (top row) and CD16^{-} (middle row) NK cells and the CIM301-8 binding (bottom row). Human NK cells were incubated with increasing concentrations of anti-MUC1 (CIM301-8; defucosylated Fc-tail). Increasing concentrations of defucosylated CIM301-8 anti-MUC1 antibody incubated with primary human NK cells only for 4h. Binding of the MUC1 antibody was determined using flow cytometric analysis labeled anti-human IgG antibodies. (**B**) Overlay histogram of CIM301-8 binding via the Fc tail on NK cells with increasing CIM301-8 concentrations. (**C**) Representative overlay histogram of CD16 expression on NK cells after incubation with CIM301-8 antibody. In all panels, one representative sample is shown.

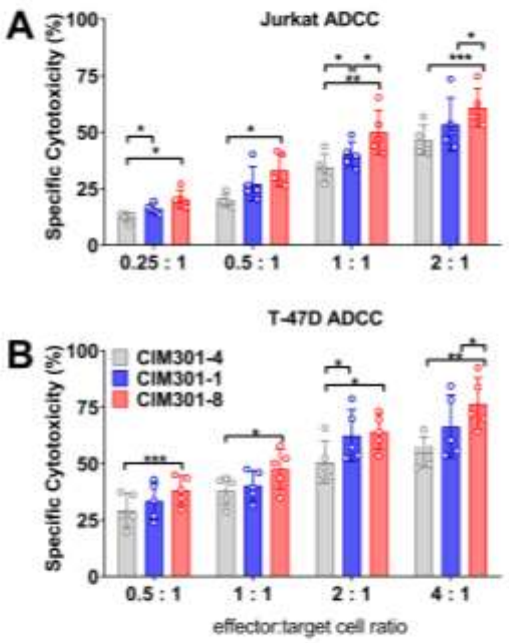

Figure S4. Humanized anti-MUC1 antibody enhances NK cell cytotoxicity via ADCC. (**A**) Quantification of antibody-dependent cellular cytotoxicity against Jurkat cells at different E:T ratios and in the presence of the indicated antibody at 1µg/mL after 4h of co-culture. Bars indicate mean ± SD, dots are 5 individual NK cell donors from independent experiments. (**B**) As A, with T-47D as tumor cells. Statistical analysis using two-way ANOVA plus Tukey's multiple comparisons test. Not significant (n.s.); $p < 0.05$ (*); $p < 0.01$ (**); $p < 0.001$ (***).

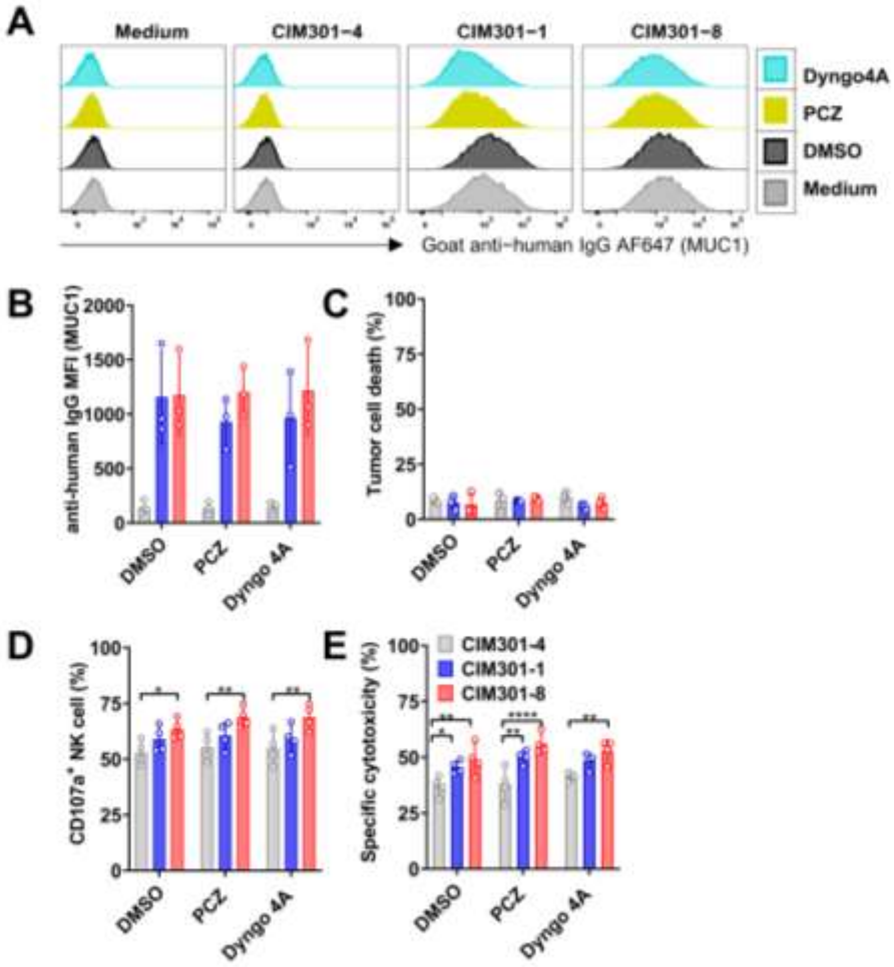

Figure S5. The endocytosis inhibitors PCZ and Dyngo4A neither upregulate the antigens on the tumor surface nor enhance the ADCC of Jurkat cells. (**A**) Overlay histograms of flow cytometric analysis of MUC1 epitope expression on Jurkat tumor cells in the presence of endocytosis inhibitors using regular (CIM301-1) or defucosylated (CIM301-8) anti-MUC1 antibodies or an irrelevant control antibody (CIM301-4). Anti-human IgG antibodies were used to detect antibody binding on tumor cells. Endocytosis inhibitors were dissolved in DMSO, here used as a negative control. One representative sample is shown. (**B**) Flow cytometric quantification of MFI of MUC1 expression levels on Jurkat cells after treatment with endocytosis inhibitors as described in (**A**). Differences between control antibody (CIM301-4; grey bars) and anti-MUC1 antibodies (regular CIM301-1 in blue and defucosylated CIM301-8 in red) were all statistically significant with $p < 0.05$ or smaller. (**C**) Viability of Jurkat tumor cells after incubation with anti-MUC1 antibodies with or without endocytosis inhibitors. Pooled data from 3 independent experiments performed at different timepoints. (**D**) Fraction of CD107a$^+$ degranulating human NK cells in co-cultures with Jurkat tumor cells at an effector/target ratio of 1:1 in the presence of anti-MUC1 antibodies and endocytosis inhibitors. NK cells and tumor cells were incubated for 4 h, with endocytosis inhibitors (5 µM PCZ, 30 µM Dyngo4A and 0.1% (*v*/*v*) DMSO) added during the last hour. (**E**) Antibody-dependent NK-cell mediated cytotoxicity against Jurkat tumor cells. Experimental setup as in (**D**). Panels (**D**) and (**E**) show pooled data from 4 independent experiments with different donors, performed at different time points. Differences between groups were determined using two-way ANOVA with Tukey's multiple comparisons test. Not significant (n.s.); $p < 0.05$ (*); $p < 0.01$ (**); $p < 0.0001$ (****).
